# EUS-guided rendezvous fistula creation for complete anastomotic stenosis after low anterior resection

**DOI:** 10.1016/j.vgie.2025.06.001

**Published:** 2025-06-17

**Authors:** Pavlos Kaimakliotis, Nicole Saur, Galen Leung

**Affiliations:** 1 Division of Gastroenterology, University of Pennsylvania Perelman School of Medicine, Philadelphia, Pennsylvania, USA; 2 Advanced Endoscopy Department, University Hospital, San Antonio, Texas, USA; 3 Division of Colorectal Surgery, University of Pennsylvania Perelman School of Medicine, Philadelphia, Pennsylvania, USA

## Abstract

**Background and Aims:**

Complete luminal stenosis at the colorectal anastomosis after low anterior resection is a rare adverse event.

**Methods:**

We present a novel case with rendezvous via a diverting ileostomy and a retrograde EUS-guided formation of a new colorectal anastomosis for recanalization.

**Results:**

A 52-year-old previously healthy man presented with rectal bleeding and was found to have locally advanced (T3N1) adenocarcinoma. The patient received neoadjuvant chemoradiation and then underwent an uncomplicated low anterior resection with a diverting loop ileostomy. During surveillance sigmoidoscopy 6 months later, complete stenosis of his anastomosis was seen, and he was referred to advanced endoscopy. He underwent dual-operator colonoscopy and ileoscopy with confirmation of complete stenosis of the anastomosis. Using EUS, we deployed a lumen-apposing metal stent for de novo colorectal fistula formation. The patient was discharged home and has since undergone successful takedown of his ileostomy. He has remained without recurrent anastomotic narrowing on routine follow-up.

**Conclusions:**

Although complete stenosis of colorectal anastomosis is rare, we argue for the role and safety profile of EUS-guided lumen-apposing metal stent insertion given the ability to distend the proximal aspect of the colon with water to create an accessible target for fistula creation, which offers a minimally invasive alternative to major surgery.

Luminal stenosis at the colorectal anastomosis after low anterior resection has been described, although complete stenosis is a rare adverse event.^1^ Rates of anastomotic strictures have been as high as 10% in studies, although complete stenosis is exceedingly rare,^2^ with risk factors including a less-than-2-cm distal surgical margin. The management of benign colorectal anastomotic strictures is well described, and a variety of devices have been shown to be effective, including balloon dilation; in more severe cases, electrocautery knives and indwelling stents can be used as an alternative to surgery.^3^^,^^4^ The use of a rendezvous technique via diverting ileostomy has been previously described, although no other studies have demonstrated de novo formation of a new anastomosis for a complete stricture with electrocautery-enhanced lumen-apposing metal stent (LAMS) placement, to our knowledge.^5^^,^^6^

Complete stenosis after low anterior resection typically involves a more invasive surgical management,^7^^,^^8^ which entails a transrectal surgical stricturoplasty. We present a novel case using 2 operators with rendezvous via a diverting ileostomy and a retrograde EUS-guided formation of a new colorectal anastomosis for recanalization.

A 52-year-old previously healthy man presented with rectal bleeding and was found to have a proximal rectal lesion consistent with a locally advanced (T3N1) adenocarcinoma. The patient received neoadjuvant chemoradiation and then was referred to a colorectal surgeon and underwent an uncomplicated low anterior resection with a diverting loop ileostomy. During his surveillance flexible sigmoidoscopy 6 months later, his primary surgeon noted complete stenosis of his anastomosis, and he was thus referred to advanced endoscopy for further management.

The patient received propofol sedation per anesthesia with a plan for rendezvous-assisted procedure given aforementioned findings. With an upper endoscope (GIF-HQ190; Olympus, Tokyo, Japan) in a retrograde approach from the anal side, a complete stenosis of the colorectal anastomosis was seen (Video 1, available online at www.videogie.org). The area was gently probed with a long 0.035-inch guidewire, which would not pass. Contrast was injected that again did not reveal any luminal patency. A second operator then advanced a pediatric colonoscope (PCF-H190; Olympus) in an anterograde approach down the efferent limb of the diverting loop ileostomy and eventually to the proximal end of the colorectal anastomosis, which was not patent from the proximal end either (Figs. 1 and 2). Contrast was injected again, which demonstrated no luminal patency. We were able to identify the light from the alternate operators' endoscopes on either side of the anastomosis (Fig. 3). However, because of concern for interposing vessels before fistula formation, the retrograde gastroscope in the rectum was removed and exchanged for a curvilinear array echoendoscope (GF-UCT180; Olympus) to perform an EUS evaluation. There were no significant interposing vessels visualized. Sterile water was flushed from the pediatric colonoscope to distend the lumen and create a large target for fistula creation. Under EUS guidance, a 19-gauge access needle was used to puncture the proximal sigmoid area and coil a long 0.035-inch guidewire under fluoroscopic, endosonographic, and direct endoscopic (from the anterograde rendezvous operator's viewpoint) guidance. The needle was exchanged for a cautery-enhanced LAMS deployment system and again under endosonographic, fluoroscopic, and direct endoscopic guidance a 15-mm × 10-mm LAMS was deployed successfully (Figs. 4 and 5). Contrast was injected, confirming luminal patency. The total procedure duration was 120 minutes.

**Figure 1:**
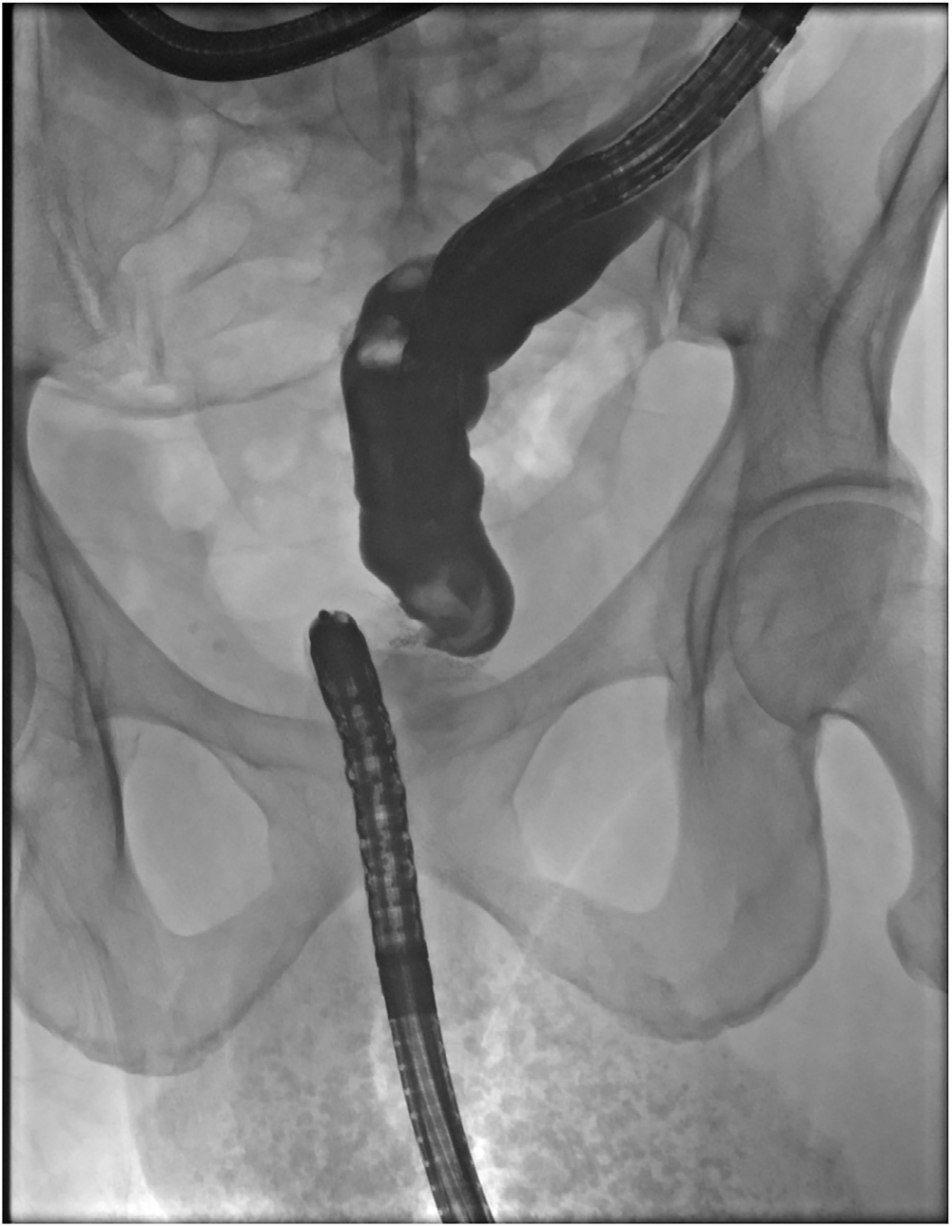
Complete luminal stenosis on enterogram without luminal patency.

**Figure 2:**
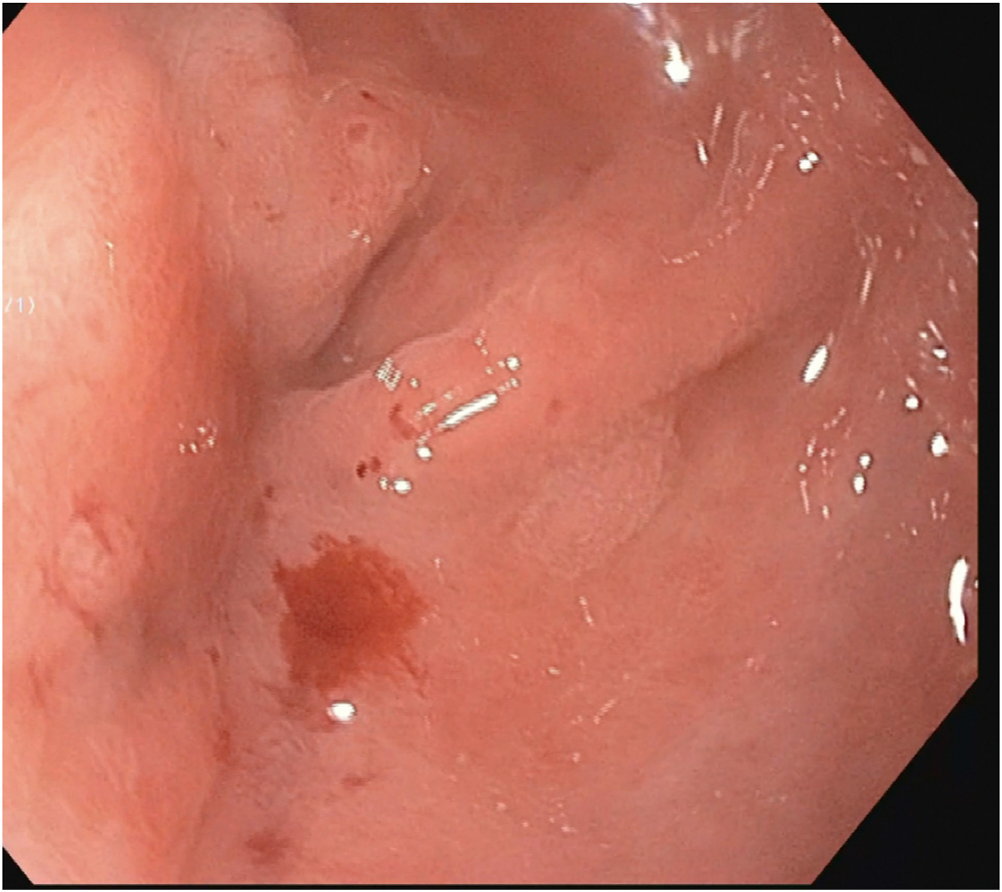
Complete stenosis of rectal anastomosis.

**Figure 3:**
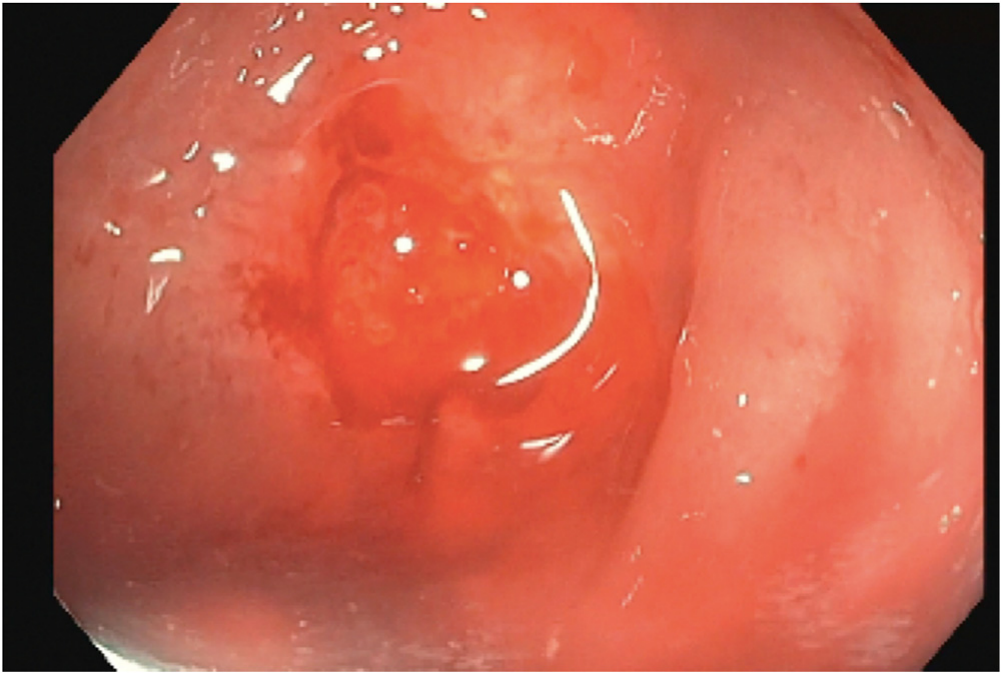
Complete luminal stenosis with light shining through from alternate operator's endoscope.

**Figure 4:**
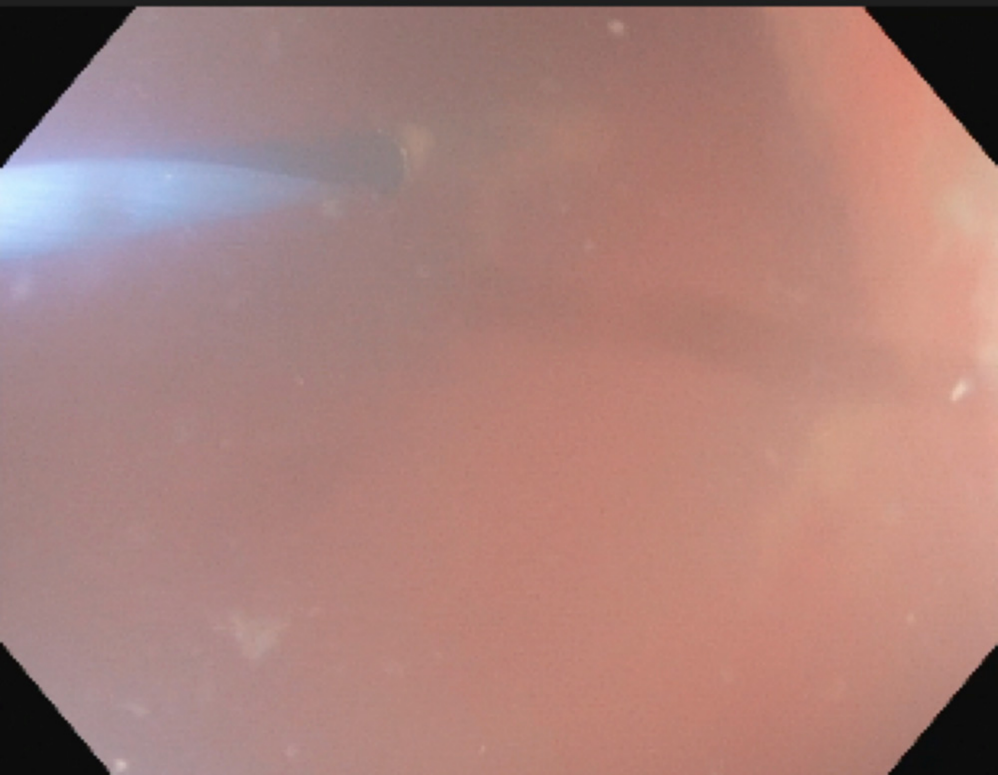
Endoscopic view from proximal aspect of anastomosis of guidewire insertion.

**Figure 5:**
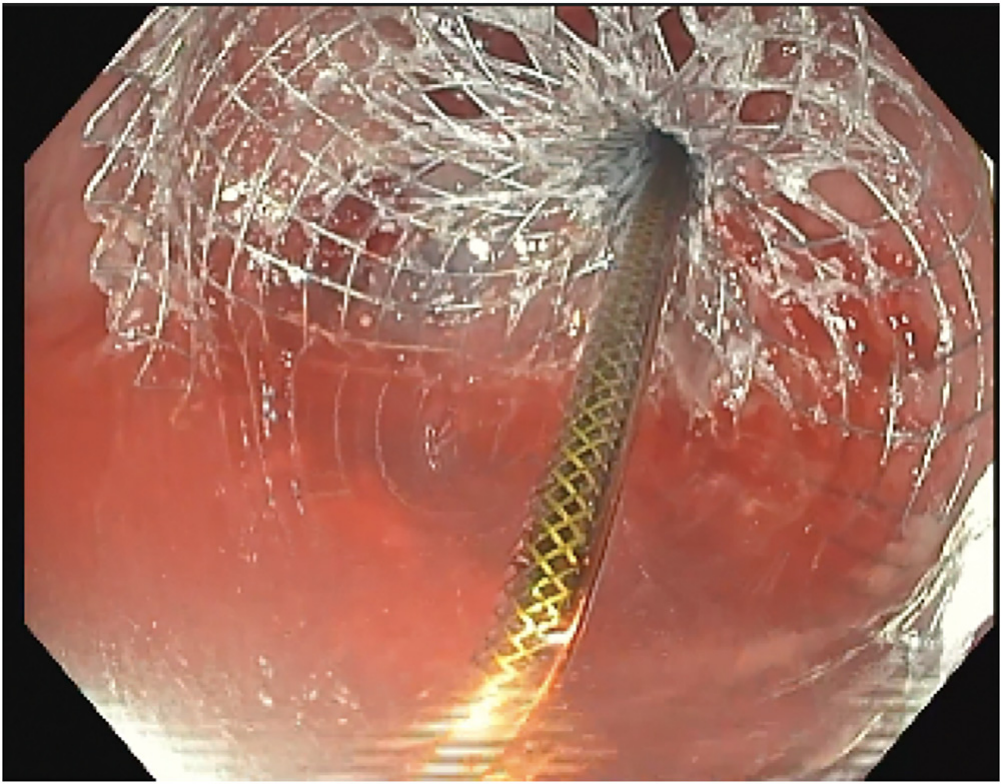
Endoscopic view from proximal aspect of anastomosis of lumen-apposing metal stent insertion.

The patient was discharged with antibiotic prescription for outpatient use and returned 3 weeks later for exchange to a 20-mm × 10-mm LAMS to further dilate the colorectal fistula. A 20-mm LAMS was not chosen on index procedure as the result of concern for inadequate space for distal flange deployment in the patient's sigmoid colon. After a total LAMS dwell time of about 2 months (to allow for a more permanent fistula to form), the patient’s prosthesis was removed, and he underwent elective ileostomy takedown without any postoperative adverse events (Fig. 6). The patient has remained without recurrent anastomotic stenosis on routine follow-up over the last 5 months and has had normal bowel function.

**Figure 6:**
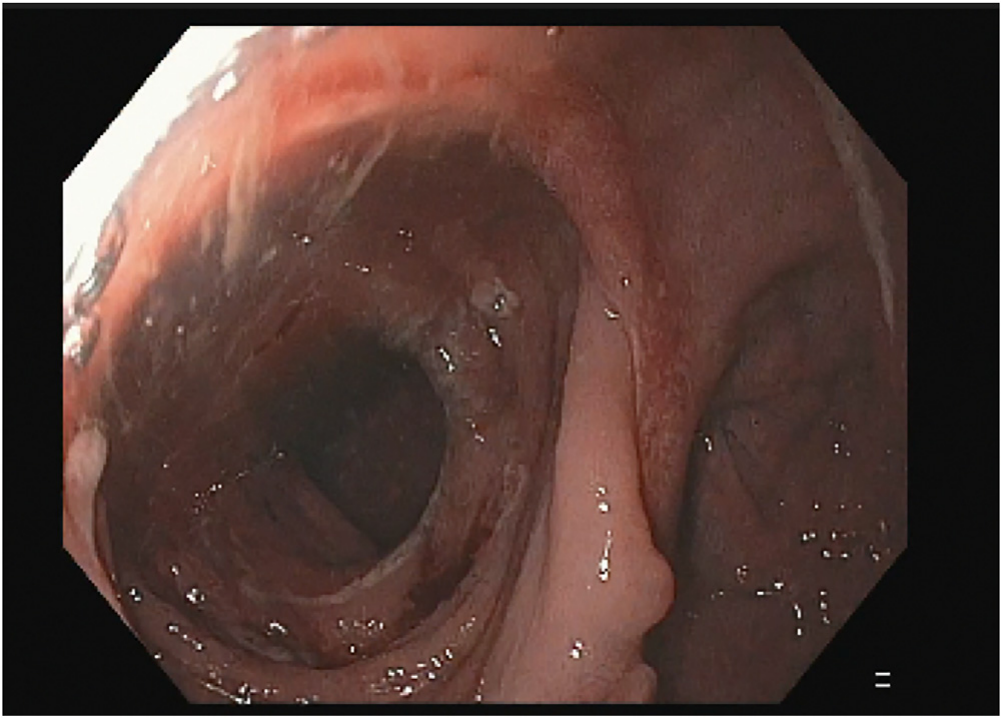
Follow-up flexible sigmoidoscopy after removal of the lumen-apposing metal stent with patent anastomosis.

Although complete stenosis of colorectal anastomosis is rare, we argue for the role and safety profile of EUS-guided LAMS insertion, given the ability to visualize any interposing vessels before fistula formation and to distend the proximal aspect of the colon with water to create an accessible target for fistula creation, which offers a minimally invasive alternative to major surgery. In addition, the LAMS allows for continued patency of the colorectal fistula before ileostomy reversal and also mitigates the risk of migration, given the lumen-apposing flanges. We do emphasize that this procedure should only be offered at tertiary centers after multidisciplinary discussion with the primary surgeon (such as performed in our case). Also, an earlier intervention postdiverting ileostomy is preferred, given that the longer the duration of an ileostomy is, there is a greater risk for more severe diversion colitis and potential colonic stricturing, making an anterograde colonoscopy technically difficult.

## Patient Consent

The patient in this article has given written informed consent to publication of their case details.

## Disclosure

The following authors disclosed financial relationships: G. Leung: Consultant for AI Medical, Boston Scientific, and Steris and Vice President of Mirai Medical. All other authors disclosed no financial relationships.

## Supplementary data

Video 1 Video case report illustrating a case of complete colorectal anastomosis stenosis, treated by EUS-guided colorectal fistula formation via dual-operator rendezvous LAMS insertion.
